# Can dogs reduce stress levels in school children? effects of dog-assisted interventions on salivary cortisol in children with and without special educational needs using randomized controlled trials

**DOI:** 10.1371/journal.pone.0269333

**Published:** 2022-06-15

**Authors:** Kerstin Meints, Victoria L. Brelsford, Mirena Dimolareva, Laëtitia Maréchal, Kyla Pennington, Elise Rowan, Nancy R. Gee

**Affiliations:** 1 School of Psychology, University of Lincoln, Lincoln, Lincolnshire, United Kingdom; 2 Department of Psychology, School of Science, Bath Spa University, Bath, Somerset, United Kingdom; 3 Center for Human-Animal Interaction, School of Medicine, Virginia Commonwealth University, Richmond, VA, United States of America; Memorial University of Newfoundland, CANADA

## Abstract

Prolonged or excessive stress negatively affects learning, behavior and health across the lifespan. To alleviate adverse effects of stress in school children, stressors should be reduced, and support and effective interventions provided. Animal-assisted interventions (AAI) have shown beneficial effects on health and wellbeing, however, robust knowledge on stress mediation in children is lacking. Despite this, AAIs are increasingly employed in settings world-wide, including schools, to reduce stress and support learning and wellbeing. This study is the first randomized controlled trial to investigate dog-assisted interventions as a mediator of stress in school children with and without special educational needs (SEN) over the school term. Interventions were carried out individually and in small groups twice a week for 20 minutes over the course of 4 weeks. We compared physiological changes in salivary cortisol in a dog intervention group with a relaxation intervention group and a no treatment control group. We compared cortisol level means before and after the 4 weeks of interventions in all children as well as acute cortisol in mainstream school children. Dog interventions lead to significantly lower stress in children with and without special educational needs compared to their peers in relaxation or no treatment control groups. In neurotypical children, those in the dog interventions showed no baseline stress level increases over the school term. In addition, acute cortisol levels evidenced significant stress reduction following the interventions. In contrast, the no treatment control group showed significant rises in baseline cortisol levels from beginning to end of school term. Increases also occurred in the relaxation intervention group. Children with SEN showed significantly decreased cortisol levels after dog group interventions. No changes occurred in the relaxation or no treatment control groups. These findings provide crucial evidence that dog interventions can successfully attenuate stress levels in school children with important implications for AAI implementation, learning and wellbeing.

## Introduction

Prolonged exposure to stressors, including academic stresses, can cause adverse effects on learning, behavior, health and wellbeing in children cross-culturally and over their lifespan, hence, it is important to prevent and reduce stressors in schools [1–9]. To counter stress-related negative effects, support needs to be provided and effective interventions are required (see [10] for recent overview).

Several types of stress-alleviating interventions have been explored in schools. For example, effects on cortisol levels were investigated using an information-based intervention which taught adolescents about stress [11]. Other interventions employed yoga [12], mindfulness [13], meditation [14] or physical activity with cognitive engagement [15] or teaching style interventions [16]). Results show overall mixed effects (see [17] for systematic review and critical analysis of methods employed).

Studies of human-animal interaction (HAI) and animal-assisted interventions (AAI) have found beneficial effects on health and wellbeing in adults and children (for recent systematic reviews see [18–23], for comprehensive overviews of recent research, please see [24–28]). Some studies have shown promising effects on stress reduction with lowered cortisol levels in adults and children [e.g. 29–35], while other studies have shown mixed evidence or minor effects in children [36, 37].

Critical voices have identified that studies in this field often lacked scientific rigor [38–41] and systematic research and randomized-controlled trials to establish a robust knowledge base are scarce [41]. However, despite the lack of an established evidence base, animal-assisted interventions (AAIs) are already employed in many educational settings world-wide and their use continues to increase rapidly (e.g. [41–43]). Thus, there is an urgent need for reliable and valid assessments of the potential effects of animals on children’s stress levels, wellbeing and academic ability to inform current practice.

As it is unknown if typically developing children and those with special educational needs (SEN) benefit in the same way or differently from AAI, we address this knowledge gap. The effects of dog-assisted interventions on children’s physiological stress responses will be reported for two cohorts–neurotypical children and children with SEN. It has also been questioned what settings and types of AAI may be most successful and cost-effective to implement [43], hence it is vital to investigate the efficacy of small group versus individual AAI intervention sessions. This has not been investigated for AAI before, and the current research integrates this question. To contribute to improved quality of studies in the field of AAI, we employed randomized controlled trials.

### Stress and learning

Adults and children are affected by stress [2, 4, 5, 7–10]. While all aim to maintain a state of equilibrium, they are challenged by internal and external adverse factors. As an individual faces a stressor [44], the autonomic nervous system (ANS) is rapidly and firstly activated through the hypothalamus. This evolved adaptive response results in epinephrine, a hormone and neurotransmitter (also known as adrenaline), to be released by the adrenal glands and some neurons into the blood stream to prepare the body for important fight-or-flight responses. This results in a series of physical changes such as increase in an individual’s respiratory rate, heart rate, blood pressure and increased blood sugar. If an individual is exposed to a more prolonged stressor (e.g., a few minutes), then hormonal changes occur such as the secretion of glucocorticoids through the hypothalamic-pituitary-adrenal axis (HPA) and the primary end product of the HPA is cortisol [45].

Cortisol can therefore be used to assess a person’s stress levels long term (chronic), or in relation to specific events (acute) with changes in acute cortisol levels typically visible after about 20–30 minutes [46, 47]. The collection of free cortisol derived from saliva in children is fairly easy to obtain, is less intrusive than other methods of collection and is relatively stable [46–48], (for wider and critical discussion see [17]). It needs to be highlighted that there is variability within and across samples; cortisol typically follows a circadian rhythm during which cortisol increases before waking and gradually decreases over the course of the day, typically reaching its lowest levels in the evening [49–51]. In addition to changes across the diurnal curve, the HPA axis also shows large intra-and inter-individual variations in response to stress [52]. During acute stress, wider changes take place in the central nervous system (CNS), which includes activation of various pathways relating to adaptive functions such as arousal, vigilance and focused attention whilst inhibiting functions such as eating and growth [44]. Overall, salivary cortisol is accepted as reliable biomarker in social science research [e.g., social psychological research into interpersonal conflict, social support and rejection, for detailed overview see 46].

In addition to the effects of stress on physiological function, stress can affect areas of cognitive processing located in the pre-frontal cortex such as attentional control, executive function, and memory [53–56]. It is important to emphasize that excessive or prolonged activation of the stress response systems has negative effects on learning, behavior, and health across the lifespan [1–4, 9, 10]. It is widely accepted that educational achievement can also be affected by stress associated with mental health disorders in children and young people [2–4]. In their interdisciplinary framework of stress and wellbeing, Sigfusdottir, Kristjansson, Thorlindsson & Allegrante (2016) [5] analyse the effects of multiple biological, social and environmental stress factors experienced during specific developmental periods, and cumulatively over time.

Stress levels seem to affect children with special educational needs, for example with Autism Spectrum Disorder (ASD), differently from their neurotypical peers [57, 58]. Higher cortisol levels than in their peers were reported in response to novel situations [e.g. 57https://www.ncbi.nlm.nih.gov/pmc/articles/PMC3885342/ - R47], school integration [58, 59], and with growing self-awareness of their lack of social competence [60]. Social situations with peers can also result in enhanced cortisol response [61], and higher stress levels have also been found in children with more complex needs and in those with growing maturity [62].

In light of the adverse effects of early life stresses and negative childhood experiences and their significant consequences over the lifespan, the quest of the National Scientific Council on the Developing Child (2015) [1] on closing the “Science-Policy Gap” (p.5) to prevent and reduce stressors, provide support and implement intervention, especially in children with mental health problems and SEN, appears even more urgent.

### Animal-assisted interventions in school children

Animal-assisted interventions (AAI) have shown beneficial effects on children’s socio-emotional and cognitive functioning, memory and behavior. Studies have found beneficial effects of AAI in schools, for example, students paid more attention to the teacher when a dog was present [63, 64], and they showed better adherence to instructions and fewer irrelevant choices and errors [65–67], see also recent overviews [18, 28]). The term animal-assisted intervention (AAI) is used here as overarching term to include interventions in general. These include targeted activities, therapy with a trained therapist or a specific educational programme which are described more specifically under the terms animal-assisted activities (AAA), animal-assisted therapy (AAT) and animal-assisted education (AAE) (see Fine [24]) for overview).

Why might dogs have such beneficial effects on humans? From an evolutionary viewpoint, humans have a long-shared history with dogs, originating from a mutually beneficial relationship in which humans provide food, shelter and safety for the pet while the pet dog contributes to physiological health and psychological wellbeing, provides social-emotional support and safety and also acts as social facilitator [68, 69]. Studies of biological mechanisms underlying the human-animal bond and its stress-reducing effects have identified physiological indices for arousal and affiliative behaviors, e.g., lower cortisol and higher oxytocin levels after interacting with a pet as well as lowered blood pressure, reduced skin conductance and lower heart rate [70–72] and [73] for overview. Similar results were found for recovering from a stressor [e.g. 74]. Furthermore, reduction of anxiety and increases in desirable social interactions were confirmed in children, healthy adults and adolescents and those with mental health problems [75, 76].

The ability of AAIs to enhance concentration, attention and motivation and reduce stress levels may be conducive to effective learning and performance with the animal’s presence creating a positive social atmosphere [26, 44, 63, 64], and for reduction in stress levels in University students [33]. An integrative approach combining biophilia, neurobiological processes, attachment and caregiving to pets seems most useful to explain the resulting human-animal relationships, their development and physiological and endocrine basis [26, 72]. The biopsychosocial model [77–79] captures and unites these varied aspects and mechanisms into one holistic model allowing for dynamic and complex interactions between biological, psychological and social factors.

However, despite such encouraging results, there is a notable lack of research to assess effects of AAI on stress in school children incorporating not only self-reported stress levels, but the collection of cortisol as independent physiological measure [18]. So far, only four studies have included the collection of salivary cortisol in assessing effects of dogs in school children. Investigating the role of dogs as social support before, during and after a stressor occurred, Beetz, Kotrschal, Hediger, Turner, Unvas-Moberg & Julius (2011) [34] reported in an exploratory study that children with insecure-avoidant or disorganized attachment patterns in a (real) dog group showed significantly reduced cortisol levels during and after stressor occurrence in comparison with a toy dog group and human social support. They concluded that the dog effectively moderated children’s levels of stress after the stressor had occurred. Beetz, Julius, Turner & Kotrschal (2012) [35] extended this work and again found similar lowered cortisol levels in the dog group. Schretzmayer, Kotrschal & Beetz (2017) [36] investigated children’s reading performance and physiological effects of a dog intervention. They reported mixed results with children in both the dog and no-dog conditions showing no differences in cortisol during their first test session but higher levels in the dog group at the second test session. The authors concluded that children were more aroused with the dog present and discuss the potential motivational effect of the dog on children’s performance through activation of the appetitive system which includes the limbic system and is associated with dopamine function related to positive affective states. They also noted that behavioral video data showed less nervous movements and less talking in the dog condition suggesting a calming effect. Finally, Kertes et al. (2017) [37] investigated the effect of pet dogs on children’s perceived stress levels and cortisol responses before and after a stress test with and without a pet dog present. While cortisol changed over time, it did not differ significantly depending on experimental condition, but interestingly, lower cortisol was associated with more child-initiated pet contact under stressful conditions.

As the inclusion of animal-assisted interventions in schools becomes increasingly popular [18, 28], and as self-report measures do not seem as reliable as independent physiological measures [34, 35], it is important that further research is carried out to gain a more complete picture of the effect of animals on children’s physiological measures.

While it is interesting to see the mediating effect of dogs after exposure to a specific short-term stressor, with AAI being used in schools already, it is vital to establish if AAI can moderate stress children experience in normal school settings over the school term.

It is also important to establish whether dog interventions lead to differential stress reductions in different cohorts, for example, in children with and without special educational needs.

A further aspect relevant to the implementation of such interventions in school settings is whether they are as effective when carried out with a small group of children as opposed to individually. Group interventions could ensure cost efficiencies for educational settings. If effective, this would also mean less working time for dog and dog handler, and hence improved animal welfare for dogs involved in AAI. This has other important implications as one-to-one and group interventions may have different dynamics. For example, an intervention carried out individually is likely to be more intense and can be more focused towards a specific child, whereas a small group intervention involves social dynamics and peer interactions which can act as social support [80–83]. This knowledge is needed to enable basic choice of cohorts and application type–currently, due to lack of research, these are chosen at random or according to practical availabilities by the setting or AAI provider.

The current project addresses this lack of research by systematically investigating the effects of dog-assisted interventions on salivary cortisol in typically developing children and in children with special educational needs, both in groups and individually using randomized controlled trials.

Baseline measures of salivary cortisol in 8-10-year old children were collected at the beginning and end of a school term (about 6 weeks’ duration) in both mainstream and special education needs schools, and acute cortisol was collected with children in mainstream schools. Employing randomized controlled trials (RCT), children took part in either dog-assisted intervention, relaxation intervention, or no treatment control conditions. These were carried out either individually or in small groups. The current study is part of a larger, longitudinal, randomized controlled trial systematically examining the effects of dog-interventions on school children’s academic performance, social and emotional wellbeing and measured physiological changes (Lincoln Education Assistance with Dogs; https://lead.blogs.lincoln.ac.uk/ [17, 18].

In line with previous research (e.g. [34–36]) a reduction in stress was predicted after dog interventions, and therefore lowest cortisol levels when comparing the dog intervention group to a relaxation intervention and a no treatment control group. It was predicted that relaxation interventions would hold an intermediate position between the no treatment group and the dog intervention, with moderate reductions in cortisol levels compared to the no treatment control group. Due to the absence of research on SEN versus typical populations, or group versus individual interventions, we compared typically developing children with those with SEN in group and individual interventions. We expected both cohorts to benefit from AAI and investigated if group or individual interventions had benefits. The current study was able to demonstrate benefits in both populations and can highlight when individual or group interventions worked best.

## Materials and method

### Participants

Children were recruited through four mainstream and seven special educational needs schools in Lincolnshire and Gloucestershire, UK. Before the study started, a priori power calculations were carried out to determine sample size (GPower 3.1.9) [84]; to obtain statistical power at the recommended .80 level for our analyses (alpha at .05, we required a minimum of 40 children per cohort for cortisol measures before and after interventions (3 conditions, 2 repetitions) and a minimum of 36 children for acute cortisol measures (3 conditions, 3 repetitions). Due to the repeated measures (up to 3 test times), we overrecruited where possible to avoid attrition.

In Study 1, 105 children aged 8–9 years in publicly-funded mainstream schools were tested (N = 54 boys, 51 girls; mean age = 8.9 years, *SD* = 0.39 years; range = 8.2 to 10.1 years). Of these, baseline cortisol samples were collected successfully both before and after intervention from N = 90 children (mean age 8.4 years, *SD* = 0.52), N = 43 boys (mean age 8.4 years, *SD* = 0.55) and N = 47 girls (mean age 8.4 years, *SD* = 0.49). The remaining fifteen children could either not provide sufficient amounts of cortisol, were absent during collection, or samples were removed due to contamination as determined by the Anglia Ruskin Labs, UK. Samples of acute cortisol (directly before and about 30 minutes after intervention sessions 1, 4 and 8) were collected from children (N = 47) from the dog and relaxation intervention groups only (mean age 8.9 years, *SD* = 0.43; range = 8.2 to 10.07 years); N = 20 boys (mean age 9.0 years, *SD* = 0.47), N = 27 girls (mean age 8.9 years, *SD* = 0.40).

In Study 2, 44 children with special educational needs were able to provide baseline cortisol (mean age 9.8 years; SD = 0.79; age range 8.3–11.4 years; N = 6 girls (mean age 10.1, SD = 1.17), N = 38 boys (mean age 9.8, SD = 0.71). Diagnoses and characteristics included Autism Spectrum Disorder (ASD) (N = 8), Attention Deficit Hyperactivity Disorder (ADHD) (N = 13), ASD and ADHD (N = 7), Down Syndrome (N = 1), other learning difficulties (e.g., profound and/or multiple learning difficulties, global developmental delay) (N = 8), unknown diagnosis (N = 7) as parents did not provide this information. Previous research has often recruited children with a prevailing main diagnosis such as ASD or mainly children with high-functioning autism. The current approach ensured maximum inclusivity of children with comorbid conditions such as severe intellectual disabilities who have historically been under-represented in research [85], including in the field of AAI.

All children were in school full-time. All researchers and dog handlers had enhanced police checks and researchers were highly experienced in carrying out research with children in schools.

### Dogs and handlers

Twenty-three different dogs and their handlers (N = 21) took part in the interventions on a volunteer basis. Most volunteers (N = 19) and their dogs were members of Pets as Therapy, a UK-registered charity providing animal-assisted therapy within community settings, and one dog handler was associated with Gloucestershire Therapy Dogs Nationwide, and dogs were insured via these charities. One dog handler was independent and separate insurance as well as separate dog assessments were obtained. Dogs were aged between 2 and 10 years and all healthy. All had passed additional assessments by independent dog behaviour specialists on their suitability to work with children. Dogs included: 1 Greek Hare-Hound, 2 Cavalier King Charles Spaniel and Miniature Poodle crossbreeds, 1 Labrador and miniature Poodle crossbreed, 2 German Short-Haired Pointers, 2 Miniature Schnauzers, 3 Labradors and 1 Labrador crossbreed, 2 Tibetan Mastiffs, 1 Border Terrier, 1 Scottish Terrier, 1 Lurcher, 1 Clumber Spaniel, 1 Yorkshire Terrier, 1 Pekingese, 1 Smooth Collie, 1 Cocker Spaniel and 1 Golden Retriever.

### Procedure

#### Informed consent

All parents/guardians provided informed written consent for their child to take part in the research and to provide saliva samples for the processing of cortisol. Children also gave assent before each intervention-session and cortisol collection. Children and parents were aware that children did not have to take part and that they were free to stop at any time.

All dog handlers provided consent to take part in the assessments and in the study. Dogs were monitored throughout the study for potential signs of wanting to withdraw, they also were free to retreat at any time.

#### Safety training and familiarization

Prior to intervention sessions, all children took part in safety training on understanding dog body language and safe behavior with dogs—this included an interactive presentation followed by a question/answer session. Children also took part in a “do’s and don’ts” activity. This took approximately 2 minutes to do and aimed at setting clear boundaries for behavior around the dogs during all sessions. In addition to reducing the potential risk of any incidents, this also ensured that children understood the dogs’ welfare needs and the requirement to uphold the dogs’ needs at all times.

Children were familiarized with the dogs prior to intervention in order to eliminate potential novelty effects. Familiarization sessions took place in the week preceding intervention, with approximately 30 minutes exposure to each dog in small groups. Children were introduced to the dog by the handler and given some general information such as breed, sex, age, likes and dislikes. Children were then encouraged to ask questions in order to gain familiarity with each dog. Children were allowed to pet the dog as the dog was led around the group by the handler to greet the children, if the handler and the researcher agreed and if the dog did not indicate any stress signalling. All children remained seated during these sessions.

The handler was briefed by the researcher if any children had anxieties concerning meeting the dog. Any child worried about meeting the dog did not need to take part–but, if they wanted to, was purposely seated next to the researcher and the greeting process was child-led. No child was made to stroke a dog or interact with any dog if they did not feel comfortable in doing so, and the handler did not approach them with the dog unless they requested it (see also [86] for detailed guidelines).

#### Interventions

Stratified randomisation was used to place children in the different intervention groups. This method ensured that we did not confound dog ownership, socio-economic status or children’s academic ability with intervention condition. Testing was carried out in waves with 1/3 of participants in the dog group, 1/3 in the relaxation control group and 1/3 in the no treatment control group to avoid potential effects of seasonal affective disorder.

*Individual and group interventions*. Children took part either in individual or in small group interventions (up to 7 children). All intervention sessions lasted for 20 minutes.

*Dog-assisted intervention*. The researcher and the dog handler were present during the whole intervention and children were supervised at all times by the researcher while the dog handler took responsibility for the dog. Each dog intervention began and ended with the child greeting the dog (as described above) as advised by the dog handler and time for petting the dog if appropriate—this phase of active contact lasted roughly 5 minutes. The next and central part of the session was based on the dog with children learning facts about the dogs from the handler, watching the dog, talking about and interacting with the dog to some extent. This was child-led and sessions varied somewhat in verbal content depending on questions children asked about the dog, and it lasted approximately 10 minutes. The last part of the session was ‘saying goodbye’, and again a chance to pet the dog–if dog, dog handler and researchers were in agreement (5 minutes).

*Relaxation intervention*. Relaxation sessions had a similar structure to the dog intervention sessions with approximately the first 5 minutes of more active relaxation (wriggling fingers, toes, etc.), then 10 minutes more quiet relaxation while listening to a story, and finally again a similar active part (5 minutes). Sessions were run by the researcher. In the sessions, children were asked to lie down on a yoga mat and two different recordings from Enchanted Meditations for Kids [87] were played. Version 1 consisted of a Jellyfish relaxation and version 2 consisted of a Butterfly relaxation. The two separate recordings were played alternately over the length of the 8 sessions: each child being exposed to each recording 4 times. Recordings were merged using Audacity 2.1.2 (1991) [88] in order that the sessions ran back-to-back without a silence not to disturb the relaxed state of the child and ensuring that the session ran within the allocated timescale of 20 minutes.

*No treatment control group*. Children assigned to the no treatment control condition took part in their regular class lessons.

#### Animal welfare

A specific protocol was devised to ensure dogs’ welfare and a risk assessment tool was created prior to carrying out the study and we strictly followed the protocol. All dogs were familiarized to the classroom and the school prior to their scheduled sessions. Dogs always had access to clean water and were able to go for a walk if the handler felt they needed it. Dogs had a bed that was their safe space and children were taught not to approach the dogs if they chose to go there. If dogs became tired or were showing any signs that they were no longer willing to take part [89], the sessions were ended. Dogs did not work more than 2 hours with some dogs only working for 1 hour. The scheduled time depended on the dog and their handler’s availability.

#### Cortisol collection

All saliva collection, handling and storage were carried out in accordance with Salimetrics LLC Saliva Collection and Handling Advice (2015) [90]. Additionally, as it is widely recognized that collection of cortisol can be challenging in school children and others [91, 92], we carried this out to a strict protocol and adhering to clear best practice guidelines [17] to minimize any potential confounding variables or contamination of samples. Collection of salivary cortisol was carried out using the passive drool method using Cryovials (3.5ml) from Salimetrics. All samples were assigned a unique, anonymized bar code provided by Salimetrics and paired with a child. No child details were included with any samples and so were anonymized at all times. Children rinsed their mouth with water and waited around 3 minutes before providing the saliva sample. After this waiting period, children were instructed to drool into the 3.5 ml cryovial until approximately 1ml of saliva was collected. Children were asked to hold the vial at the bottom and not touch the top.

Children in mainstream schools were given verbal instructions. To ensure children with special needs understood the task, verbal explanations were given to children, while others needed to imitate the drooling process, or, more direct one-to-one supervision was enabled to collect the saliva as the children collected it in their mouth before drooling into the vials. As some children with special needs were anxious around new people, teachers and teaching assistants helped with the collections of the samples after having read the protocol, or with the researcher being present. In all cases, it was ensured that children did not touch the inside of the vial or the inside of the vial caps. Each cryovial was then immediately capped and placed in between ice blocks in a pathology bag to keep all samples cool until they could be frozen in the lab at -20^o^.

**Fig 1:**
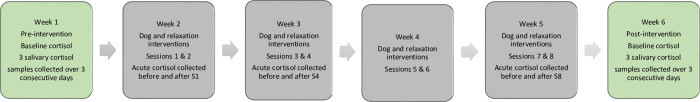
Timeline in overview. Timeline for collection of salivary cortisol samples, and timing of interventions.

*Cortisol collection before and after intervention period*. Children’s mean cortisol levels were measured before and after the 4-week intervention period. Pre-intervention period samples were taken over three consecutive days (one per day) before intervention began, and a further three post-intervention samples were taken over three consecutive days immediately after the intervention period finished–this is as cortisol levels can vary and is advised best practice [17, 92] (Fig 1).

For statistical analyses we used the mean cortisol levels before interventions (calculated from the first 3 measures) and compared this with the mean cortisol levels after 4 weeks (calculated from the last 3 measures). Therefore, we obtained two cortisol level means, one before and one after the intervention period of 4 weeks; these cortisol samples were not collected on the same day as the interventions.

In contrast, our acute cortisol testing occurred just before and after an intervention session on the same day (see below under ‘acute cortisol collection”).

Samples were taken in the mornings between 9.30 and 10.15am. Collecting first samples after 9.30am was to ensure that no teeth brushing, no food or sugary drink intake had occurred in the previous 30 minutes after entering the school setting, in addition to no vigorous exercise having taken place prior to samples. Three further consecutive samples were to be obtained on three further consecutive days after the last intervention session. In mainstream schools 82% of children provided 3 samples before and 86% after intervention (see Table 1).

**Table 1:** Overview of samples provided.

Cohort	Number of samples	Children providing samples before intervention period started (N)	Children providing samples after end of intervention period (N)
Mainstream schools	3	74	78
	2	15	16
	1	3	4
SEN schools	3	21	17
	2	12	15
	1	11	12

Samples provided by children per cohort.

Sample numbers were lower in SEN schools due to other school, private or medical commitments, or sickness. In some cases, this was also due to the large distance between schools. Due to the nature of the SEN schools and children’s anxiety, it was not possible to simply send a replacement researcher, therefore less than 3 samples were used so as not to upset children or disturb their routine.

*Acute cortisol collection*. Children in the mainstream schools provided acute cortisol before and after intervention sessions. Acute salivary cortisol was collected before interventions and 25–30 minutes after the end of interventions for sessions 1 (start), 4 (middle session) and 8 (end); sessions took place at the same time in the day. Passive drool was used as described above. Children in SEN schools were not able to provide acute cortisol before and after interventions sessions.

*Sample treatment*. Samples were transported to the University around an hour after collection but no later than 4–6 hours after collection, where they were stored in a locked room in a freezer at -20°C for up to 5 working days before being shipped to Salimetrics LLC for storage until analysis. Samples were transported in line with UN3373; were triple packed between icepacks, labelled “Human Saliva Samples-Biological Substance Category B- UN3373” and contained a manifest of all barcode samples enclosed. Samples were independently analysed by the Biomarker Analysis Laboratory at Anglia Ruskin University, Cambridge, UK using Salimetrics Salivary Cortisol ELISA kits. Samples were assayed for salivary cortisol using a high sensitivity enzyme immunoassay (Salimetrics Europe Ltd). 10% of samples were assayed in duplicate. Concentration of single samples, or first duplicates, was 1μg/dL and second duplicates 2 μg/dL. If the coefficient of variation for the concentration between the duplicate repeats was greater than 15%, then saliva samples were re-run, unless absolute values between the first and second samples were within 0.03μg/dL. All samples were destroyed immediately after analysis by Anglia Ruskin Labs, UK.

*Ethics*. This research was approved by the University of Lincoln Research Ethics Committee (SOPREC) and are in line with BPS Ethics guidelines. In addition, this project was reviewed and approved by the MARS Research Review Board and the WALTHAM Animal Welfare and Ethical Review Board.

### Statistical analysis

Data was inspected for normality and transformed as inspection of the pre-intervention data using the Shapiro-Wilk statistic revealed that assumptions of normality for the mean cortisol measure were violated (W = .886 *p* < .001), with skewness of 1.551 (SE = .209) and kurtosis of 3.419 (SE = .416). After data was log transformed (Log10), data tended to normality and parametric tests were used to analyse the mean cortisol measures. Cortisol results between cohorts are presented first to establish potential cohort differences. Repeated measures ANOVAS were then carried out on Condition as between-subjects factor (dog intervention, relaxation intervention, no treatment control) and Time as repeated measure (before and after intervention) per cohort (typically developing children in mainstream schools (Study 1) and children with SEN (Study 2)). Analysis was next run separately for group and individual testing conditions. It is important to note that for all intervention conditions specific predictions, calculated with planned comparisons, were of core interest as it was predicted specifically that children in the dog intervention would show *least or no* increase in cortisol over time while we did expect cortisol increases in the no treatment control group and to some extent in the relaxation group. Significance testing follows the usual p-value criterion of smaller than .05 for significant results, and for planned comparisons smaller significance levels were used employing Bonferroni-Holm corrections. Descriptive statistics reported reflect the raw data [93]. Statistical analysis was carried out using Statistica 12 as well as IBM SPSS, version 26.

## Results

### Salivary cortisol levels in mainstream versus SEN cohorts

**Fig 2:**
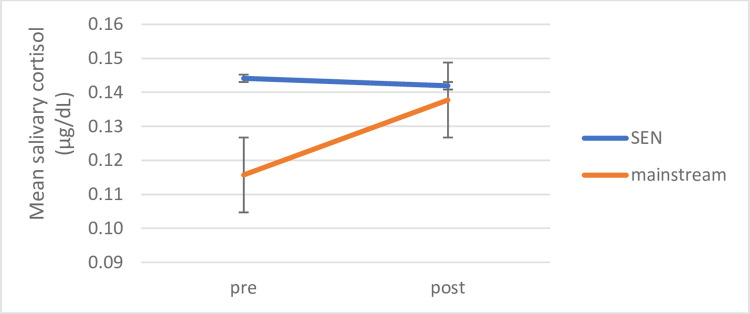
Significant cohort differences pre-post intervention. Differences between cohorts pre- to post intervention, figure shows mean salivary cortisol (μg/dL), error bars indicate standard error of mean.

An initial Time (pre/post) x Cohort (Mainstream, SEN) analysis of variance was calculated to assess possible differences in cortisol before and after interventions between those children attending mainstream schools and those attending SEN schools. There were no significant main effects, but a significant interaction for Time and Cohort (*F* (1,132) = 4.616, *p* = .034, *ŋ*_*p*_^*2*^ = .034) demonstrated significant differences between the cohorts in children’s salivary cortisol. Post-hoc paired samples t-tests revealed that children attending special educational needs schools did not show an overall difference in cortisol measures between pre (*M* = .1454 μg/dL, *SD* = .06) and post-intervention (*M* = .1429 μg/dL, *SD* = .09) overall; (t (43) = .308, *p* = .760, *d* = .046). This contrasts with children in mainstream schools who showed a significant overall increase in cortisol between pre (*M* = .1157 μg/dL, *SD* = .06) and post-intervention (*M* = .1377, μg/dL, *SD* = .08) (t (89) = -3.402, *p* = .001, *d* = .36) (see Fig 2 below).

Further one-way analyses of variance showed that there was also a significant difference in cortisol measures between both cohorts before interventions began (*F* (1,133) = 7.151, *p* = .008, *ŋ*_*p*_^*2*^ = .051) (mainstream Pre-*M* = .1157 μg/dL; *SD* = .06; SEN pre-*M* = .1454 μg/dL, *SD* = .06). This difference was no longer present after interventions (*F* (1,133) = .100, *p* = .752, *ŋ*_*p*_^*2*^ = .001) (mainstream post-*M* = .1377 μg/dL, *SD* = .08; SEN post-*M* = .1429 μg/dL, *SD* = .09). To investigate these differences relative to intervention-condition and session-type, each cohort was analysed separately next.

### Study 1

#### Effects of AAI on mean salivary cortisol levels in children in mainstream schools

A one-way analysis of variance was conducted to assess whether pre-intervention period cortisol means were significantly different at the beginning of the study, based on the school of the child. A significant effect of School was returned (*F* (3, 89) = 2.908, *p* = .039, *ŋ*_*p*_^*2*^ = .092), however, post hoc t-tests were not significant, demonstrating that in the current data differences in children’s pre-intervention cortisol levels between pairs of schools were not significant. Therefore, the school that children attended was not considered a significant contributor to differences in cortisol measures. A further one-way analysis of variance assessed whether there were significant differences in children’s pre intervention period cortisol means based on dog-ownership before interventions began. No significant difference was found between those children who owned a dog (*M* = .1080 μg/dL, *SD* = .05) and those who did not (*M* = .1220 μg/dL, *SD* = .064) (F (1, 89) = 1.508, *p* = .223, *ŋ*_*p*_^*2*^ = .017).

A repeated measures ANOVA of Time (pre/post intervention) x Condition (dog, relax, control) was conducted with planned comparisons investigating the core questions of intervention-specific effects. A significant main effect for Time (F (1, 87) = 11.167, *p* = .001, *ŋ*_*p*_^*2*^ = .114) revealed increases in cortisol overall. No main effect for Condition, or interaction between Time and Condition reached significance. In order to investigate the predicted differences of the dog and relaxation interventions and no treatment conditions before and after intervention, planned comparisons were conducted using paired samples t-tests. These revealed the following differences: children in the no treatment control group showed a significant and the highest increase in mean cortisol levels after the 4-week period with medium effect size (*t* (19) = -2.749, *p* = .013, *d* = .62) (pre *M* = .1108 μg/dL, *SD* = .03; post *M* = .1379 μg/dL, *SD* = .05) (significance level with Bonferroni-Holm correction p = .0167). Children in the relaxation condition just missed a significant increase in mean cortisol levels when comparing before and after intervention cortisol levels and, as predicted, these are somewhat less in intensity with lower effect size (*t* (35) = -2.334, *p* = .025, *d* = .39) (pre *M* = .1196 μg/dL, *SD* = .08; post *M* = .1554 μg/dL, *SD* = .11) (significance level with Holm-Bonferroni-Holm correction p = .025).

**Fig 3:**
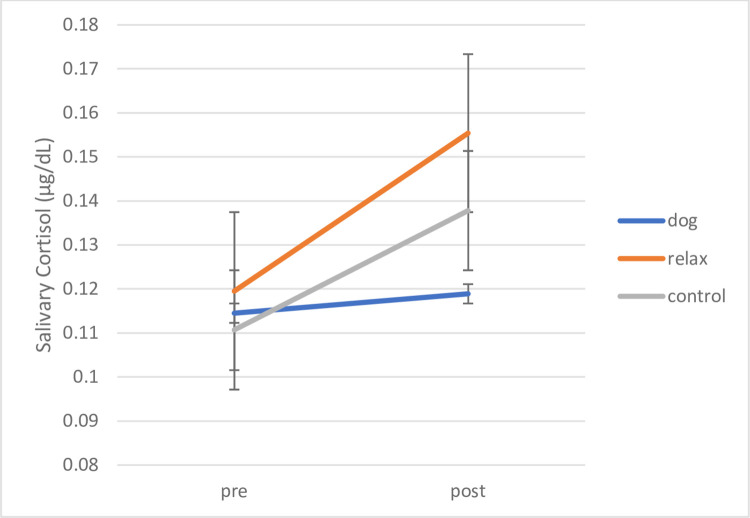
Mainstream cohort: Mean pre-post salivary cortisol by intervention condition. Mean pre-post salivary cortisol levels shown by intervention condition. Error bars indicate standard error of mean.

In contrast, and as predicted, children in the dog intervention exhibited no significant change in mean cortisol levels between measures before intervention at the start of the school term (*M* = .1145 μg/dL, *SD* = .05) and after intervention at the end of the school term (*M* = .1189 μg/dL, *SD* = .03) (*t* (33) = -1.272, *p* = .212, *d* = .22). This indicates that they showed no increases in stress hormone from beginning to the end of school term in the dog intervention condition only. Fig 3 illustrates this result.

#### Effects of AAI on mean salivary cortisol levels in children in mainstream schools: Individual interventions

A repeated measures ANOVA of Time (pre / post intervention) x Condition (dog, relax, control) was conducted. A significant main effect for Time was evident (F (1,56) = 9.248, *p* = .004, *ŋ*_*p*_^*2*^ = .142) with increases in cortisol overall after intervention. No main effect for Condition or interaction of Time with Condition was revealed. To assess the predicted differences between the conditions, planned comparisons with paired samples t-tests revealed the following differences between interventions conditions: in line with the previous analysis children in the no treatment control group showed the highest increase in mean cortisol levels over time with medium effect size (*t* (19) = -2.749, *p* = .013, *d* = .62) (pre M = .1108 μg/dL, *SD* = .03; post M = .1379 μg/dL, *SD* = .05); (significance level with Bonferroni-Holm correction p = .0167). Children in the relaxation condition also showed an increase in mean cortisol levels after intervention, however, while this difference shows a trend, it is less strong and misses significance (*t* (19) = -1.904, *p* = .072, *d* = .43) (pre M = .0992 μg/dL, *SD* = .05; post M = .1482 μg/dL, *SD* = .13).

**Fig 4:**
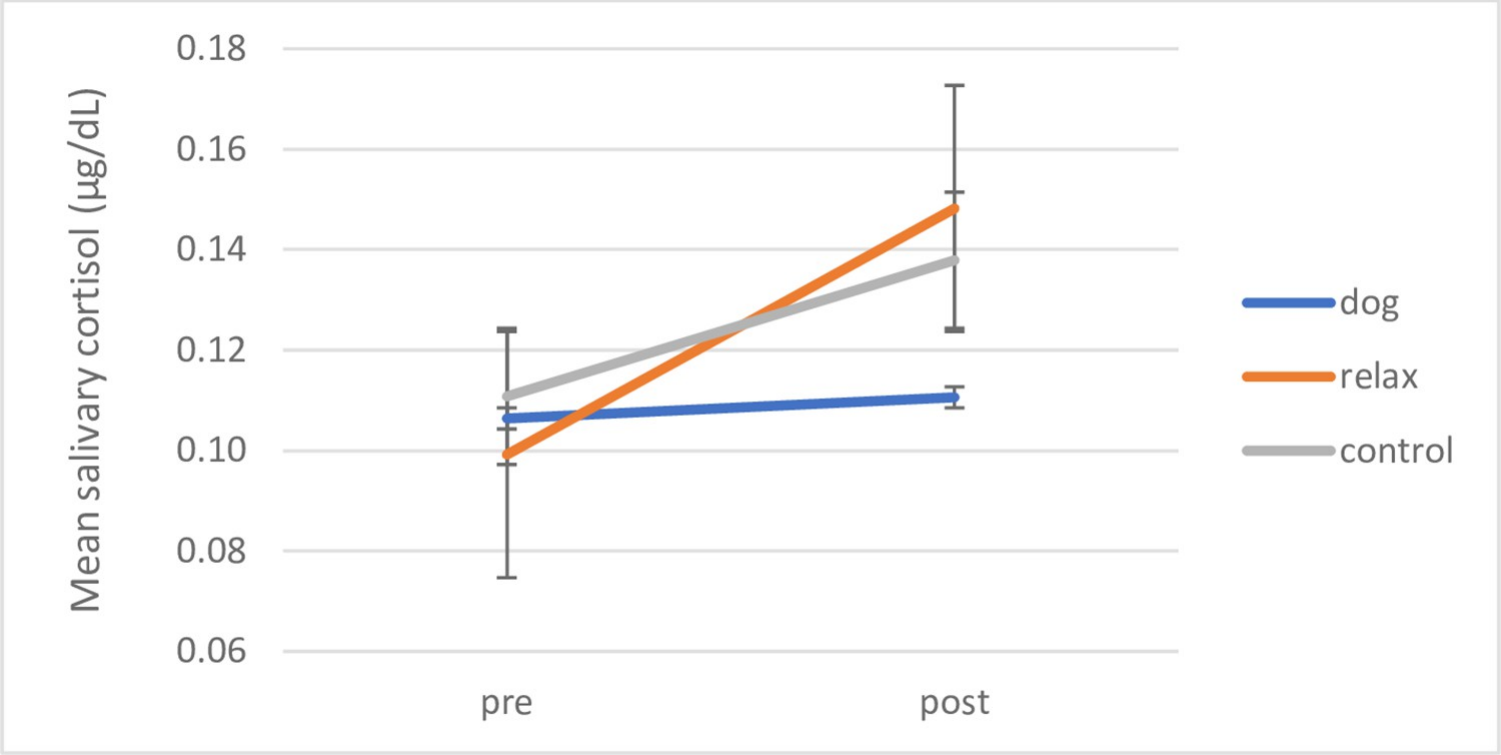
Individual interventions: Mean pre-post salivary cortisol by intervention condition (dog, relax, no treatment control). Mean pre-post salivary cortisol levels shown by intervention condition (dog, relax, no treatment control). Error bars indicate standard error of mean.

Again, children in the dog intervention exhibited no significant change at all in mean cortisol levels between measures before intervention at the start of the school term (M = .1064 μg/dL, *SD* = .06) and measures after intervention at the end of the school term ((M = .1106 μg/dL, *SD* = .02) (*t* (18) = -1.092, *p* = .289, *d* = .25)). Thus, only children taking part in the dog intervention showed no increases in stress hormone from beginning to school term end. Fig 4 illustrates this.

#### Effects of AAI on mean salivary cortisol levels in children in mainstream schools: Group interventions

A repeated measures ANOVA of Time (pre/post intervention) x Condition (dog, relax, control) was conducted for children taking part in small group interventions. A significant main effect for Time (F (1,48) = 6.535, *p* = .014, *ŋ*_*p*_^*2*^ = .120) indicated increases in cortisol overall. No other significant main effects or interactions emerged. To investigate the predicted differences between the dog intervention and other conditions, planned comparisons investigated these core questions of intervention-specific effects with paired samples t-tests.

**Fig 5:**
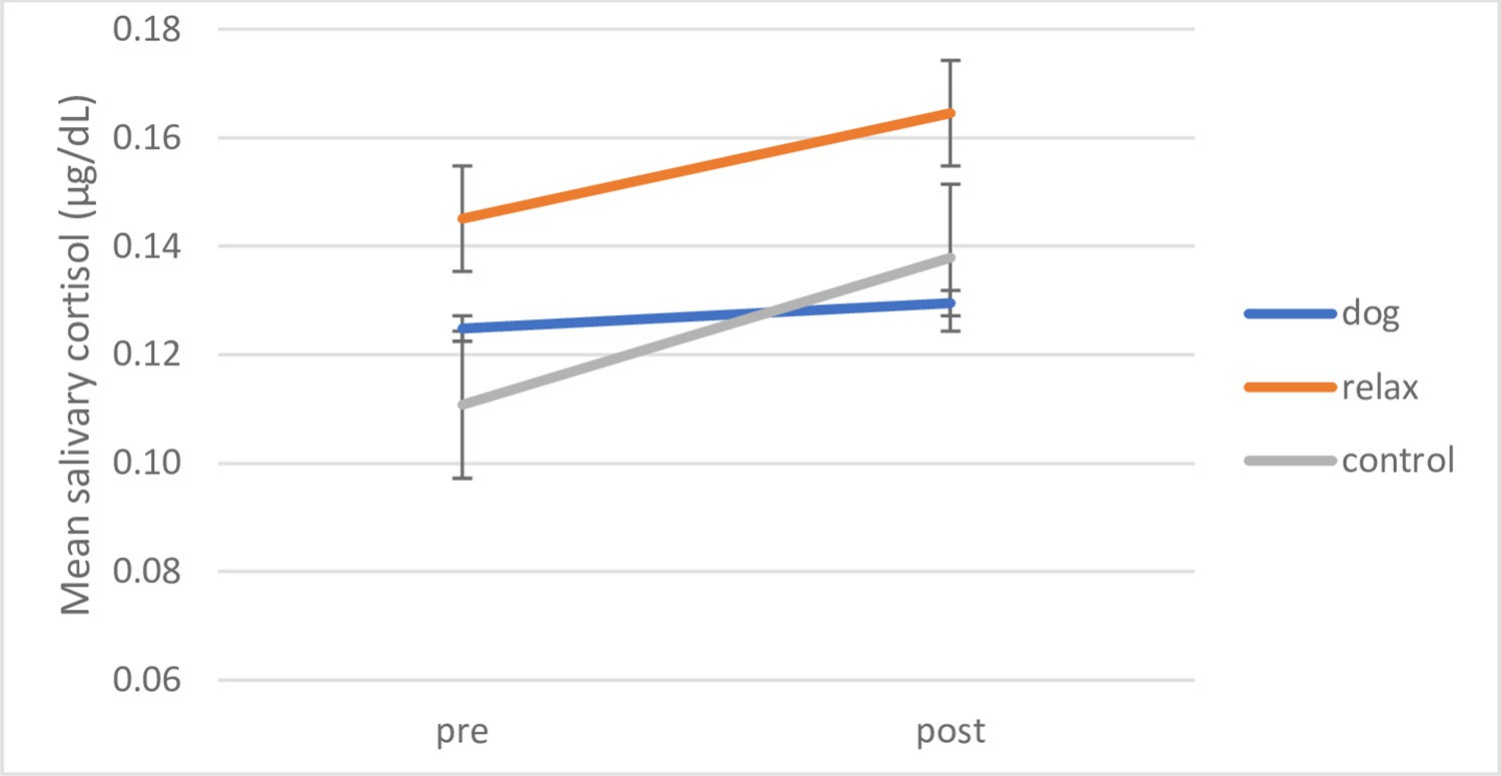
Group interventions: Mean pre-post salivary cortisol by intervention condition. Mean pre-post salivary cortisol levels shown by intervention condition. Error bars indicate standard error of mean.

A similar pattern as in the individual interventions above emerges with the highest increases occurring in the control condition as described above (p< .0167 as per Bonferroni-Holm correction). As visible in Fig 5, children in the relaxation and dog conditions showed no significant increase in cortisol means after intervention ((relax (*t* (15) = -1.326, *p* = .205, *d* = .33), (pre M = .1451 μg/dL, *SD* = .10; post M = .1645 μg/dL, *SD* = .09); dog (*t* (14) = -6.29, *p* = .539, *d* = .16), (pre M = .1248μg/dL, *SD* = .05; post M = .1295 μg/dL, *SD* = .04); control (pre M = .1108 μg/dL, *SD* = .03; post M = .1379 μg/dL, *SD* = .05)).

#### Effects of AAI on acute cortisol in mainstream schools: All children

**Fig 6:**
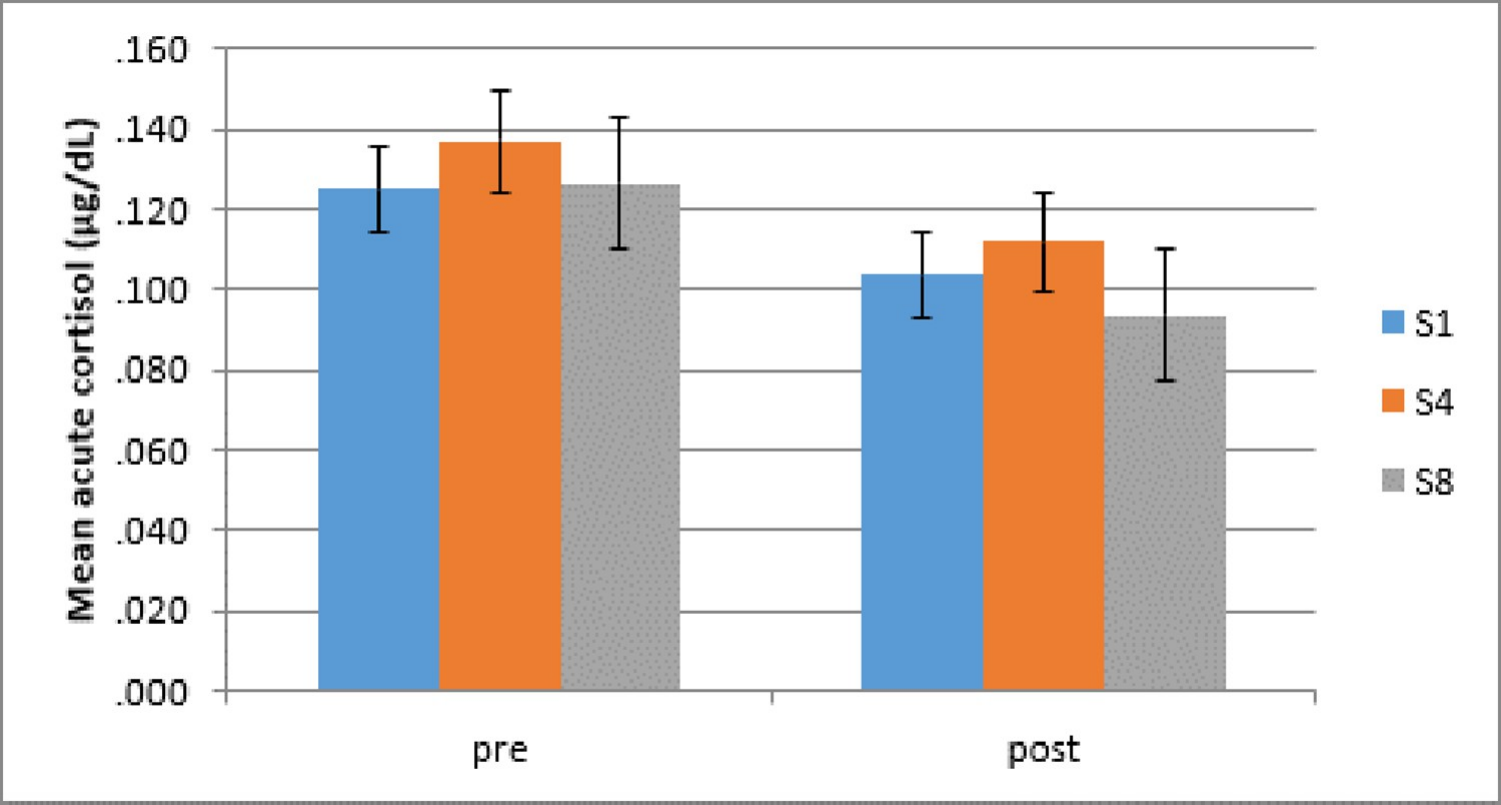
Mainstream schools: Mean acute cortisol before and after individual intervention sessions week 1, 4 and 8 (S1, S4, S8). Acute cortisol depicted before and after individual intervention sessions for week 1, 4 and 8 (S1, S4, S8). Error bars indicate standard error of mean.

Inspection of the data shows that pre-intervention cortisol measures across all time points violated the assumptions of normality. Data was log transformed (Log10), and then data tended to normality, hence parametric tests were used to analyse the acute cortisol measures. A repeated measures ANOVA of Time (pre/post intervention) x Intervention Session (week 1, 4, 8) x Condition (dog, relax) was conducted to assess the effect of AAI on children’s measures of acute cortisol. A highly significant main effect of Time (F(1, 90) = 26.532, *p* < .001, *ŋ*_*p*_^*2*^ = .371) showed a significant reduction in children’s acute cortisol after interventions. Planned comparisons with paired samples t-tests revealed significant reductions of acute cortisol after interventions for each of the intervention sessions; for Session 1 (pre: *M* = .1251 μg/dL, *SD* = .06; post: *M* = .1038, *SD* = .06), (*t* (46) = 2.903, *p* = .006, *d* = .42), Session 4 (pre: *M* = .1369, *SD* = .06; post: *M* = .1119, *SD* = .07), (*t* (46) = 3.179, *p* = .003, *d* = .46), and Session 8 (pre: *M* = .1265, *SD* = .09; post: *M* = .0936, *SD* = .04), (*t* (46) = 2.959, *p* = .005, d = .43), (significance levels with Bonferroni-Holm correction *p* = .05 (S1); .0167 (S4); .025 (S8) respectively) (see Fig 6).

No other main effects or interactions were significant. To assess if dog interventions led to the predicted lower cortisol levels compared to relaxation sessions, planned comparisons were carried out. Children in the dog interventions had a significant reduction in cortisol immediately after session 1 (t (23) = 2.646, p = .014, d = .54) with medium effect sizes (significance level with Bonferroni-Holm correction p = .0167), but not after session 4 (t(23) = 1.513, p = .144, d = .31) and session 8 (t (23) = .833, p = .413, d = .17).

**Fig 7:**
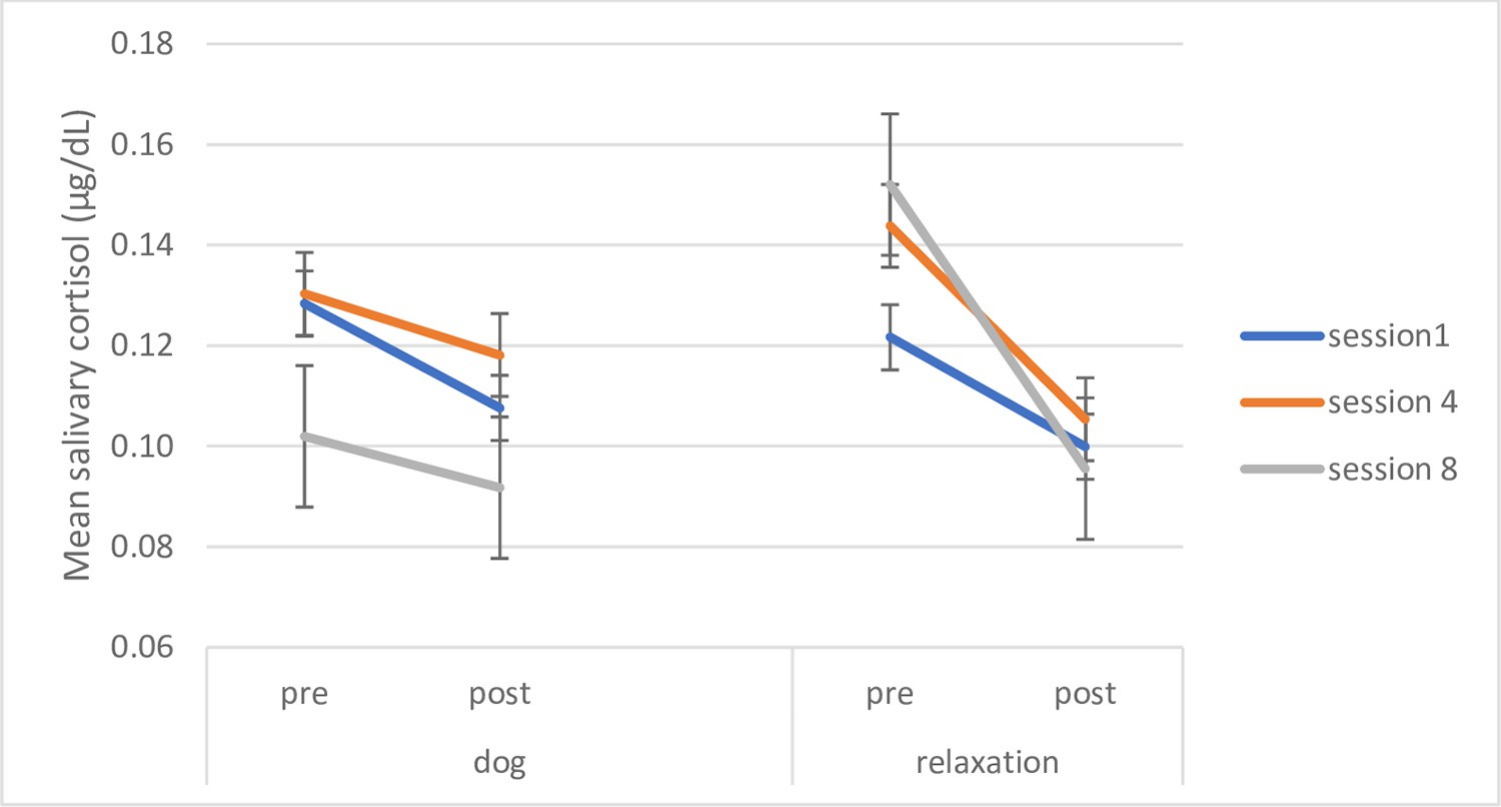
Acute cortisol: Dog and relaxation interventions at sessions week 1, 4 and 8 before and after intervention. Acute cortisol shown for dog and relaxation interventions for sessions in weeks 1, 4 and 8 before and after intervention. Error bars indicate standard error of mean.

Children in the relaxation interventions showed a contrasting pattern, with no significant reduction following session 1 (t (22) = 1.713, p = .101, d = .36), an interestingly somewhat delayed effects with significant cortisol reductions after session 4 (t (22) = 3.131, p = .005, d = .65) and session 8 (t (22) = 3.209, p = .004, d = .67) (significance level with Bonferroni-Holm correction p = .0167) (see Fig 7), both with medium effect sizes. See Fig 7 below and Table 2 for means and SD.

**Table 2:** Overview table: Acute cortisol, mainstream cohort.

Condition Session		Mean	Std. Deviation
dog	S1Pre	.1284	.061
	S1Post	.1076	.068
relax	S1Pre	.1216	.060
	S1Post	.0999	.055
dog	S4Pre	.1303	.047
	S4Post	.1181	.081
relax	S4Pre	.1438	.071
	S4Post	.1054	.063
dog	S8Pre	.1020	.043
	S8Post	.0918	.034
relax	S8Pre	.1520	.112
	S8Post	.0955	.042

Acute cortisol, mainstream cohort: Means and standard deviations per condition and intervention session (1, 4 and 8, each before and after intervention).

### Study 2

#### Effects of AAI on mean salivary cortisol levels in children in SEN schools

**Fig 8:**
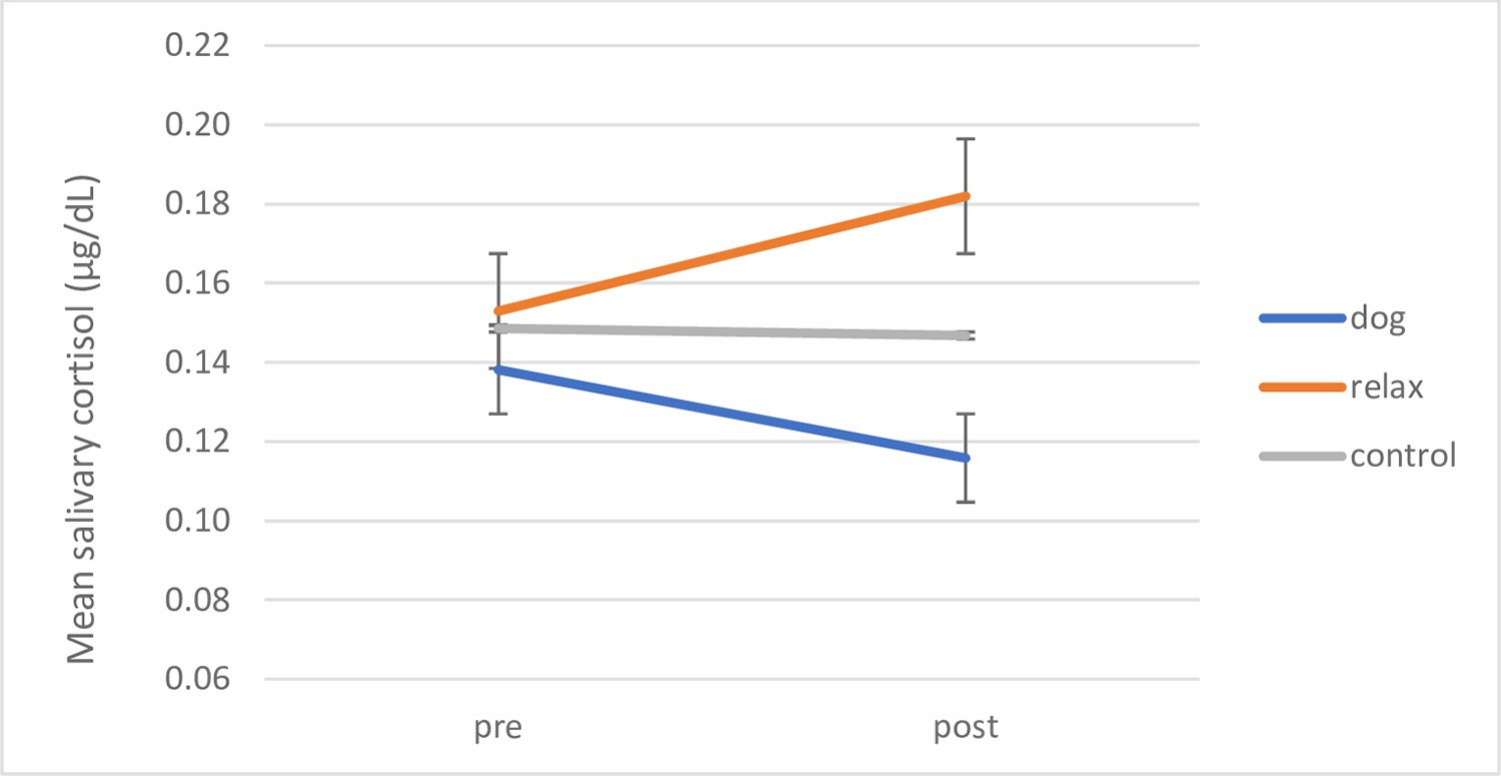
SEN cohort: Mean pre-post salivary cortisol by intervention condition. Mean pre-post salivary cortisol levels for SEN cohort shown by intervention condition. Error bars indicate standard error of mean.

A one-way analysis of variance was conducted to assess whether pre-intervention period mean cortisol levels were significantly different at the beginning of the study, based on the school the child attended. No significant effect of School was returned (*F* (6, 43) = .828, *p* = .556, *ŋ*_*p*_^*2*^ = .118), hence data for the schools were collated in further analyses. A further one-way analysis of variance assessed whether dog-ownership had an effect on cortisol before interventions began. No significant difference was found between those children who owned a dog (M = .1390 μg/dL, *SD* = .06), those who did not (M = .1505 μg/dL, *SD* = .07), and those whose dog-ownership status was unknown (M = .1431 μg/dL, *SD* = .06) (F (2,43) = .021, *p* = .980, *ŋ*_*p*_^*2*^ = .001). Note that we added a group of “unknown” here as not all parents shared the information. A repeated measures ANOVA of Time (pre/post intervention) x Condition (dog, relax, control) did not show any significant main effect (Time (F (1, 41) = .001, *p* = .977, *ŋ*_*p*_^*2*^ = .000), Condition (F (2, 41) = .994, *p* = .379, *ŋ*_*p*_^*2*^ = .046)), nor an interaction of Time by Condition (F (2, 41) = 1.382, *p* = .262, *ŋ*_*p*_^*2*^ = .063) (see Fig 8).

As we predicted specific differences in mean cortisol levels *before* and *after* the intervention period for the different intervention conditions, planned comparisons were undertaken, but changes did not reach significance in any condition (dog (*t* (17) = 1.136, *p* = .272); pre M = .1381 μg/dL, *SD* = .06; post M = .1158 μg/dL, *SD* = .04); relaxation ((*t* (10) = -.887, *p* = .239; pre M = .1529 μg/dL, *SD* = .09; post M = .1820 μg/dL, *SD* = .15); or no treatment control ((*t* (14) = .487, *p* = .634); pre M = .1486 μg/dL, *SD* = .05; post M = .1468 μg/dL, *SD* = .06). Next, further assessment of the cortisol data was carried out for each intervention type.

#### Effects of AAI on mean salivary cortisol levels in children in SEN schools: Individual interventions

A repeated measures ANOVA of Time (pre/post intervention) x Condition (dog, relax, control) was conducted to assess the effects of AAI on children with SEN taking part in individual intervention sessions. A main effect of Time (F (1, 26) = 6.224, *p* = .019, *ŋ*_*p*_^*2*^ = .193; observed power .671) showed significant increases in cortisol overall (pre-M = .1436 μg/dL, SD = .07; post M = .1629 μg/dL, Sd = .10). No main effect for Condition (F (2, 26) = .836, *p* = .836, *ŋ*_*p*_^*2*^ = .014) and no interaction of Time with Condition (F (2, 26) = 3.038, *p* = .065, *ŋ*_*p*_^*2*^ = .189) reached significance. To assess the predicted differences in mean cortisol levels based on intervention conditions, paired samples t-tests were conducted. None reached significance (dog (*t* (8) = -1.639, *p* = .140; d = .55); (pre M = .1280 μg/dL, *SD* = .07; post M = .1463 μg/dL, *SD* = .04), relaxation (*t* (4) = -1.374, *p* = .241; d = .61); (pre M = .1565 μg/dL, *SD* = .12; post M = .2413 μg/dL, SD = .21), no treatment control (*t* (14) = .487, *p* = .634, *d* = .13); (pre M = .1486 μg/dL, *SD* = .05; post M = 1468 μg/dL, SD = .06)).

#### Effects of AAI on mean salivary cortisol levels in children in SEN schools: Group interventions

A repeated measures ANOVA of Time (pre/post intervention) x Condition (dog, relax, control) revealed a main effect for Time with significant decreases in cortisol overall (F (1, 27) = 11.082, *p* = .003, *ŋ*_*p*_^*2*^ = .291) (pre-M = .1487 μg/dL, post M = .1254 μg/dL). A significant interaction of Condition with Time was also revealed (F (2, 27) = 5.619, *p* = .009, *ŋ*_*p*_^*2*^ = .294), both significant results show large effect sizes with observed power .894 and .817 respectively. To assess the predicted differences per condition, planned comparisons with paired samples t-tests revealed that children with SEN in the dog group intervention showed a highly significant *decrease* in cortisol means with a high effect size (*t* (8) = 4.157, *p* = .003, *d* = 1.39; pre-M = .1482 μg/dL, SD = .05; post M = .0853 μg/dL; SD = .02) (significance level with Bonferroni-Holm correction p = .0167); achieved power of .98.

**Fig 9:**
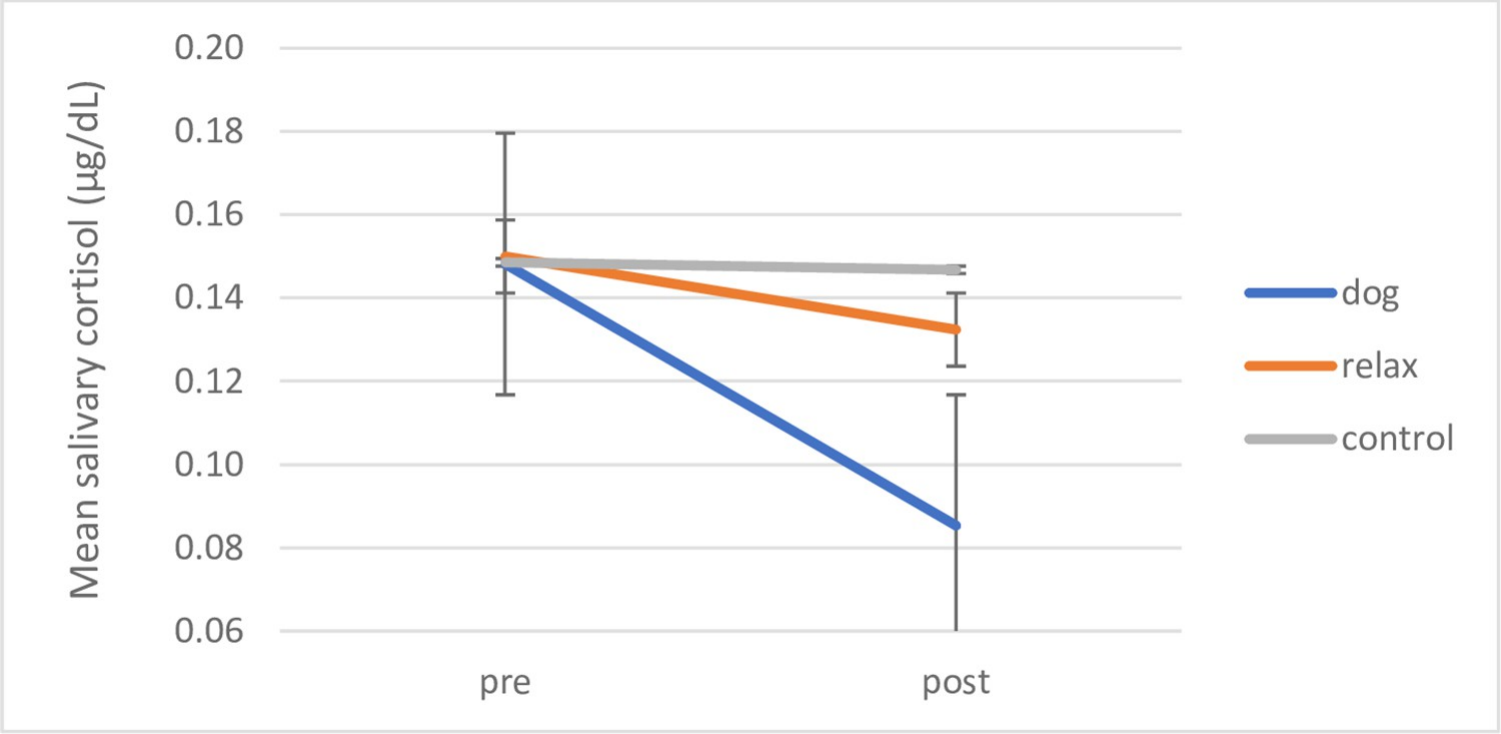
SEN cohort: Group interventions, mean pre-post salivary cortisol by intervention condition. Mean pre-post salivary cortisol levels for children with SEN by intervention condition in group interventions. Error bars indicate standard error of mean.

Children in the relaxation condition (*t* (5) = -796, *p* = .462, *d* = .33; (pre M = .1500 μg/dL, SD = .06; post M = .1324 μg/dL; SD = .05)) and no treatment control group (*t* (14) = .487, *p* = .634, *d* = .13), (pre M = .1486 μg/dL, SD = .05; post M = .1468 μg/dL, SD = .06)) showed no significant differences in cortisol levels when comparing means before and after the intervention period (see Fig 9).

## Discussion

This research is the first to demonstrate mediating effects of AAI on cortisol levels in school children over the school term. These effects were found in both, children with and without, SEN. The study also pioneers the investigation of the efficacy of individual versus group interventions.

First, we established that there were no differences in children’s cortisol levels within each cohort between different schools. There were no differences either between groups who owned a dog versus those that did not. Our study also confirmed that neurotypical children and those with special educational needs differ with respect to their cortisol levels, with children in special educational needs schools having significantly higher mean cortisol levels at study start than their typically developing peers–this adds further physiological evidence to previous research [54] and is in line with research that has found increased reactivity to stress and novel stimuli in children with autism [57–62].

Interestingly, we also found that this difference between cohorts was no longer present after intervention and towards the end of the school term–and cortisol levels of children in mainstream schools in control and relaxation groups rose over the 6-week school term to be more similar to SEN children’s cortisol levels. This is a novel and important finding in itself, as it clearly shows the effects of school stress on children’s cortisol levels–strikingly, with neurotypical children’s cortisol levels elevated at the end of the school term to levels of children with SEN. This increase is likely to be the result of the pressures children face within current mainstream educational settings as described by other research above [1–11], for example, academic pressures and limited good-quality child-care and education. While higher cortisol levels had been reported in children with SEN in response to novel situations [57] school integration [58, 59], and with growing self-awareness of their lack of social competence [60], we can now also add that stress levels rise significantly in typically developing children over the school term. Given the scientific evidence of adverse effects of stress on learning as described above, this increase in the typical population is alarming. A recent teacher survey showed an increase in stress, anxiety and panic attacks by 78% of primary schools with school leaders also reporting increased fear of academic failure (75%) and depression (55%) among their pupils in the period since 2014 [94]. The physiological data in our study evidences this change over the school term, adds to the existing evidence-base and lends much needed scientific evidence to teachers’ observations.

It should be emphasized that our measure does not represent a snapshot of data taken towards the end of an academic year nor is this in response to a singular stress event; cortisol collections and interventions were carried out as part of rolling program across school terms over the whole year, considering seasonal changes as the study started at either spring, summer or autumn terms across school settings. This result therefore captures children’s real increase in stress levels in mainstream educational settings within a typical school term. While some work on interventions of different types, from teaching interventions [11] to yoga, mindfulness and other interventions [12–17] has begun and shown mixed effects, the current results on the mediating effects of dog interventions are clearly promising and worthy of further investigation of effects of mounting pressure on school children as a consequence of educational targets and exam pressures [1–11].

Concerning the main question of this study, if AAI can help to reduce stress in school children, it was predicted that children in the dog intervention condition would show least rise in cortisol, followed by the relaxation condition, and the no treatment control group showing highest cortisol levels. As predicted, typically developing children in the dog intervention, whether individual or group interventions, did not show any significant increases in cortisol levels over the school term when comparing means before and after the intervention period. This clearly highlights the beneficial effects specifically of the dog intervention for children in mainstream schools. They did not exhibit increases in stress hormone as the control group did–the latter experienced a significant increase in cortisol levels. The relaxation group also showed an increase, albeit less pronounced. Thus, as predicted, strongest stress mediation was achieved with the dog intervention while relaxation interventions had less of a mediating effect than dog interventions.

Regarding acute cortisol before and after intervention sessions, children in mainstream schools also showed a significant reduction immediately after interventions (see also [29] and [33] for results with adults). Reduction in dog interventions occurred after the first intervention session, whilst in relaxation interventions a significant reduction occurred after sessions four and eight. In combination with the cortisol results obtained before and after the intervention period, the dog intervention showed a consistent reduction in children’s cortisol across the school term. In contrast, children in the relaxation interventions showed a significant reduction in acute cortisol, but this increased to pre-intervention levels in between each session, showing more fluctuations and less longevity than the dog interventions.

For children with SEN, who started with higher cortisol levels, it depended on whether they took part in individual or in small group interventions whether their cortisol levels stayed at the same high level, increased or decreased. Children with SEN showed increased cortisol levels at the end of term after intervention in all intervention conditions when taking part in individual interventions. However, when they took part in group interventions, those in the dog intervention group showed a striking decrease in cortisol levels, indicating a decisive reduction in stress levels. No significant decreases were evident in the relaxation or the control group. Again, this substantiates the protective effect of the dog-assisted intervention for children with SEN if carried out in a small group with other children. Group interventions may have a more intense effect on children with SEN than individual dog interventions, as they facilitated increased social interaction, in addition to feedback and support from peers who can act as social support, e.g. [35, 95]. Importantly, it must be noted that children with SEN all had some form of social, emotional and/or behavioral difficulties, so they may have preferentially benefited from a group intervention which potentially carries less social pressure than an individual intervention. It would therefore appear that the group intervention may preferentially suit the SEN cohort who present with social, emotional and behavioral difficulties. Such a facilitating effect of dogs as “social catalysts” has been reported in previous research, e.g. [68, 69] and links well to research with other participants groups (e.g., [30, 31, 33, 70, 78]. While individual and group interventions have been carried out in other areas within the health services with mixed evidence as to their efficacy, and as logistic difficulties in RCTs with individual and group interventions have been noted recently [80], it is important that future research investigates the dynamics of group interventions within AAI and the effect on cortisol and children’s social, emotional and behavioral measures further.

The current results on changes in stress levels over the typical 6-week school term are novel and complement results by others on the stress-moderating role of dogs as social support for children with insecure-avoidant or disorganized attachment patterns during or after a short-term, acute stressor occurred [34, 35]. The current study found such a stress-moderating effect in children over the longer period of a 6-week school term in mainstream school children as well as in children with special educational needs.

Our results also complement other research on children’s perceived stress levels and cortisol levels before and after a stress test [37] with lower cortisol linked to more child-initiated pet contact under stressful conditions as in our study we found lower cortisol also linked to children attending the dog group in which they were allowed to touch the dog—after consideration of safety and dog signalling [42, 86, 89], and in agreement with dog handler and researcher. Our results may well be linked to being able to touch and stroke the dog during the 20-minute intervention. The current research is limited insofar as it has not analysed the amount of opportunities to touch the dog in detail—future research and video analysis of such study data should ideally include effects of touch versus non-contact in dog interventions, where possible also in RCTs with relevant control conditions.

The current findings also add to the understanding of acute cortisol changes (i.e. effect of individual sessions on levels of cortisol) in mainstream school children. Lower cortisol levels were found in the dog intervention as well as the relaxation intervention group after interventions, but not in the no treatment control group. As children with SEN were not able to provide cortisol before and after individual intervention sessions, the current study is limited here to typically developing children in mainstream schools, but future research should explore acute cortisol with children with SEN as well as the acute cortisol gives a good insight into the direct effects of the interventions.

Typically, the number of children with SEN who are able to provide cortisol samples is limited–this was also seen in the current study. Hence, despite our best efforts, the SEN cohort is smaller in size than the mainstream cohort, and effects sizes are small in some conditions. Hence, replication with a larger SEN sample size would be desirable (if achievable).

A further limitation, linked to the currently achieved sample size in children with SEN, is that these children could not be split up into different ability groups for more detailed analysis. Future research with children with different abilities should study potential differences between such groups and further investigation may uncover that some children within a SEN cohort benefit more from interventions than others [62]. This, in turn, may lead to clearer best practice recommendations to policy-makers and stakeholders in future.

Furthermore, while the current study provided a 20-minute intervention to achieve the above results, we cannot provide further information on effects of different dosages on children. There is currently no systematic research into dosage effects and studies are needed to investigate which amount of time and contact is most effective for which group of children, and longitudinal studies could trace effectiveness over time. Finally, it may also be of interest to investigate school type and other school-related factors in more detail in future research.

Overall, the current results are an important and novel addition to the field with first evidence that dog interventions effectively moderated and attenuated children’s levels of stress over the school term, both in mainstream schools and in SEN cohorts.

The current inclusive approach ensured that children with more severe intellectual disabilities who have historically been under-represented in research [85], were included in the study to establish that benefits are generalisable to the real-life environment of special needs schools. This also enables the provision of AAI for children with severe needs who may not otherwise have appropriate interventions to enhance their development.

As only very limited research had been conducted with AAI in small groups, e.g. [57], and no comparison between group and individual intervention had been investigated within the same study so far, the current research has begun to address this knowledge gap. The current results, while necessary to be replicated, could lead to the recommendation of running small group AAIs in children with SEN if the aim is to reduce stress, while children in mainstream schools seem to benefit both from individual and group interventions.

Finally, to address why dogs might have such beneficial effects on humans, the current evidence speaks in favor of social support and facilitation [68, 69] with lower cortisol levels after intervention. Such reduction of stress levels after dog interventions may also be due to the dog creating a positive social atmosphere [e.g. 26, 34, 63, 64, 72, 73] and an integrative model like the biopsychosocial model [77–79] seems best-suited to explain the findings within one holistic model allowing for complex and dynamic mechanisms and interactions between biological, psychological and social factors.

## Conclusion

In conclusion, this research is timely and makes an important and original contribution to the field as it is the first to show that dog-assisted interventions can reduce stress levels in school children with effects lasting over the school term.

Employing RCT and integrating careful consideration of safety and welfare for all involved, we were able to discover beneficial effects of dog interventions in children in mainstream schools and children who attended special educational needs’ schools. Children in mainstream schools showed significant increases in stress hormones in the control group, while children in the dog intervention showed no such increase in stress hormone by the end of the school term having undergone either individual or group interventions with a dog.

In contrast, children with SEN benefitted most from group interventions with a dog, as evidenced by the reduction in cortisol levels by the end of the school term.

Next to these stress-moderating effects of the dog interventions, this research shows for the first time that group interventions work either as well as individual interventions (mainstream cohort) or can be more suitable with SEN cohorts as far as stress reduction is concerned. Relaxation interventions had a moderate beneficial effect only in typically developing children. Finally, these interventions were carried out with the highest safety and animal welfare standards [18, 42, 86] and with an inclusive approach to enable children to take part.

Future work will need to investigate further questions of group dynamics, social pressure, individual differences and dosage, as well as differences in children’s ability and the role of physical touch among other questions to refine advice and guidance on best practice for AAI. As this work investigated AAI effects in everyday school settings, it is innovative in integrating typical and hard to reach populations and in finding best ways to administer AAI (see also [42, 86]). In turn, this will help enable future best practice recommendations and guidance for implementation of AAI.

Overall, the current research enhances knowledge in the field of HAI and AAI showing convincing evidence of lower stress in school children due to a dog intervention and this may help change public understanding of AAI, provide a basis for changes in educational practice, including public policy change and enhance the health and wellbeing of children while providing safe and welfare-oriented interventions.
